# Metastable attunement and real-life skilled behavior

**DOI:** 10.1007/s11229-021-03355-6

**Published:** 2021-08-12

**Authors:** Jelle Bruineberg, Ludovic Seifert, Erik Rietveld, Julian Kiverstein

**Affiliations:** 1 Department of Psychiatry, Academic Medical Centre, University of Amsterdam, Amsterdam, The Netherlands; 2 Department of Industrial Design – Atlas 7.130, Eindhoven University of Technology, PO 513, 5600 MB Eindhoven, The Netherlands; 3 Department of Philosophy, Macquarie University, Sydney, Australia; 4 CETAPS Laboratory - EA 3832, Faculty of Sports Sciences, University of Rouen Normandy, Mont Saint Aignan, France; 5 Amsterdam Brain and Cognition, University of Amsterdam, Amsterdam, The Netherlands; 6 Department of Philosophy, University of Twente, Enschede, The Netherlands

**Keywords:** Metastability, Affordances, Sports science, Adaptive behavior, Attunement

## Abstract

In everyday situations, and particularly in some sport and working contexts, humans face an inherently unpredictable and uncertain environment. All sorts of unpredictable and unexpected things happen but typically people are able to skillfully adapt. In this paper, we address two key questions in cognitive science. First, how is an agent able to bring its previously learned skill to bear on a novel situation? Second, how can an agent be both sensitive to the particularity of a given situation, while remaining flexibly poised for many other possibilities for action? We will argue that both the sensitivity to novel situations and the sensitivity to a multiplicity of action possibilities are enabled by the property of skilled agency that we will call *metastable attunement*. We characterize a skilled agent’s flexible interactions with a dynamically changing environment in terms of metastable dynamics in agent-environment systems. What we find in metastability is the realization of two competing tendencies: the tendency of the agent to express their intrinsic dynamics and the tendency to search for new possibilities. Metastably attuned agents are ready to engage with a multiplicity of affordances, allowing for a balance between stability and flexibility. On the one hand, agents are able to exploit affordances they are attuned to, while at the same time being ready to flexibly explore for other affordances. Metastable attunement allows agents to smoothly transition between these possible configurations so as to adapt their behaviour to what the particular situation requires. We go on to describe the role metastability plays in learning of new skills, and in skilful behaviour more generally. Finally, drawing upon work in art, architecture and sports science, we develop a number of perspectives on how to investigate metastable attunement in real life situations.

## Introduction

How can an agent be stable enough to learn from its interactions with its environment and flexible enough to use this skill to adapt to novel situations and to remain open to a multiplicity of action possibilities? In order to deal with a complex and uncertain environment, adaptive agents are simultaneously ready for a multiplicity of possible courses of action. We use the term “skilled intentionality” to refer to the selective engagement with multiple relevant action possibilities in a concrete situation (Bruineberg and Rietveld, 2014). The agent can’t simply depend on existing patterns of engagement but must also be ready to explore for novelty or for different ways of doing things. From a dynamical systems perspective, such flexible selectivity is well captured by the notion of metastability.^1^ Metastability refers to two competing tendencies (Kelso, 1995, 2012): the tendency of the parts of the system to segregate and express their own intrinsic dynamics, and the tendency of the parts to integrate, and coordinate globally to create new dynamics. The transience that is characteristic of metastable dynamics allows the agent to negotiate between relying on existing patterns of engagement and exploring for new modes of engagement that better adapt them to the demands of their current situation.

As an example of metastability consider a skilled boxer training on a heavy bag. The boxer takes up a position in relation to the bag from which they are ready to perform multiple punches: a jab, a hook and an uppercut (Hristovski et al., 2006a, b). From this position they can easily switch between these punches. It does not matter that the heavy bag is fluctuating in unpredictable ways because the boxer is positioned in relation to the bag in such a way that they can flexibly adapt. They can respond to the particular random fluctuations of the boxing bag, both by switching between actions (jab, hook and uppercut) as well as fine-tuning each of these actions as they are performed to the specific and unpredictable fluctuations of the bag. In a real-life environment, outside of the lab, the boxer will also be ready to switch to performing many other activities, say listening to her coach about how to improve her performance, or taking an important call from the hospital on her mobile phone. Thus, at the same time as the boxer adapts her punches to the moment by moment fluctuations of the heavy bag, she is also ready to flexibly and rapidly switch to dealing with a potentially open-ended range of other possible situations.

We propose to call the metastable dynamics necessary for skilled action in real-life environments “metastable attunement”. This terminology needs some unpacking. Attunement is a concept with a history in both the phenomenology of skilled agency and ecological psychology. Dreyfus (2007) uses the word attunement to characterize the affective way in which a human is sensitive to the demands of the situation. One example of such attunement comes from Merleau-Ponty (2002/1945), who describes how in an art gallery, you may be drawn towards a vantage point on a painting from which the painting can be best seen. From too far off, you experience a felt tension, or disattunement, that can be resolved by taking a step closer. In this phenomenological sense, attunement refers to the (pre-reflectively) experienced pragmatic ``fit’’ between agent and environment, the extent to which a skilled agent is able to align its openness to the world with the demands of the situation (Rietveld, 2008; Dreyfus, 2007; Araujo et al., 2019). We will further develop this phenomenological perspective in Sect. 2 of the paper.

In ecological psychology, the term attunement is used to refer to the process of becoming sensitive to informational variables that make possible the perception of relevant affordances.^2^ In other work in ecological psychology, attunement has been characterized as a developmental process equivalent to the `education of attention’ (De Vries et al., 2015; Heft, 2001; Withagen & Van der Kamp, 2010). One marked difference between these two ecological approaches is the extent to which agents are actively selecting and creating the relations they have with their environment.

A dynamic and active understanding of attunement has been developed in the literature on ecological dynamics. Ecological dynamics is an interdisciplinary framework that combines ecological psychology with dynamical systems theory and the complexity sciences (Araujo et al., 2009; Button et al., 2020; Seifert et al., 2017). Within ecological dynamics, skilled behavior is seen as the emergence of flexible performance solutions resulting from the interplay between a skilled agent and the affordances offered in its environment. In some recent applications of ecological dynamics, the behavioral data of ecological dynamics is complemented by reports on the phenomenology of skilled action (Seifert et al., 2014a, b; Seifert et al., 2017; Rochat et al., 2019, see also Araujo et al., 2005). The point here is twofold: skilled agents actively structure their coupling with the environment and this relation to the environment is experienced.^3^

Our aim in this paper is to introduce the notion of “metastable attunement” as a bridging concept to connect ecological dynamics to phenomenology.^4^ Both perspectives—the phenomenological and the ecological dynamical—capture something important about skilled agency. Phenomenology is necessary for describing the first-person lived experience of being a skilled agent acting in a meaningful world.^5^ Ecological dynamics is also necessary for explaining skilled agency in real life settings. The integration of ecological dynamics with phenomenology allows for the investigation of skilled action outside of the lab. But while both perspectives are necessary neither will suffice on its own. There is a need for synthesis, for some means of connecting the descriptions and conceptual tools of phenomenology to the otherwise distinct vocabulary provided by ecological dynamics. We provide this bridge in our paper by developing the concept of metastable attunement.

The remainder of our paper is comprised of four sections. In Sect. 2, we introduce the Skilled Intentionality Framework. The aim of this conceptual framework is to provide bridging concepts that allow for a synthesis of ecological dynamical explanations of a skilled agent’s flexible interactions with a dynamically changing environment with first-person phenomenological descriptions of skilled agency. In Sect. 3, we provide a more detailed account of metastable dynamics in agent-environment systems. We develop our thesis that metastable attunement is necessary for dealing with the enormous variability in the situations encountered in everyday life. In Sect. 4 we explain how the balance between stability and flexibility that metastability provides allows the individual to remain poised between exploiting their existing behavioural repertoire and exploring novel possibilities for action. We suggest this poise is necessary for adapting to the particular situations encountered in real life. This completes our argument that metastable attunement is necessary for skilled action in real-life settings. Finally, in Sect. 5, we develop a number of perspectives on how to investigate metastable attunement in real life situations using art, architecture and sport science.

## The skilled intentionality framework: bridging the phenomenology and science of skilled action

There are a number of striking aspects of the phenomenology of skilled everyday activity that stand in need of explanation in cognitive science but remain little understood. First off, everything agents do with skill will be both *context-sensitive*, and situation-dependent. When visiting a library for instance it is appropriate to switch your mobile phone to silent mode, and not to make a call no matter how urgent when sitting in a silent reading area. On the other hand, making a phone call is fine when you are out of the library on the street, or when you are in the library’s café. Second, skilled behaviour is *change-in-context-sensitive*: when the fire alarm goes off, or, less dramatically, when the library closes, a skilled agent rapidly switches to the new context and the action possibilities that are appropriate now. Third, every situation is in a sense a novel and unique situation in which events unfold in subtly different ways from how they did in the past. In some cases, the situations will be rather similar (such as the same library on consecutive days), the situation might be mildly different (such as a library in a different country), or completely (such as in a society without books). Agents in exercising their skill make use of their previous history of interactions as a scaffold to understanding the present new situation. Merleau-Ponty describes this influence of past history on current first-person perception of the world in terms of an “’intentional arc’, which projects round about us our past, our future, our human milieu, our physical situation, our ideological situation, and our moral situation, or rather, that ensures that we are situated within all of these relationships” (Merleau-Ponty, 1945/2012). Past experiences manifest in how a person experiences the environment in the present and going into the future.

In what follows we seek to bridge the phenomenology of skilled action with the framework of ecological dynamics (Davids et al., 2012; Button et al., 2020; Seifert et al., 2013; Araujo et al., 2019). From this perspective, the right scale of analysis for investigating skilled action is the scale of the self-organising joint agent-environment system. The agent and environment stand in a mutual and reciprocal relation. The environment offers a rich variety of action possibilities scaled to the agent’s body given that the skills and abilities the agent develops have become attuned to them. We will henceforth refer to these action possibilities offered by the environment as “affordances” (Gibson, 1979). The environment is richly resourceful in terms of the affordances it makes available. Affordances structure and scaffold our skilled activities. They enable and structure what an agent does by functioning as constraints that limit the agent’s degrees of freedom. The agent “joins forces” with affordances in acting with skill (Ingold, 2013).

Researchers in ecological dynamics have begun to integrate phenomenological first-person data into their experimental studies. In a study of ice climbing for example, phenomenological data was used to understand why expert climbers “crossed” ice tools (Seifert et al., 2014a, 2014b). One might think that crossing ice tools is not important for ice climbing as it does not help to balance the body. However, this is not the case; expert climbers make crossing actions to explore for existing holes in the icefall that offer an economic and rapid anchorage of the ice tools (Seifert et al., 2014a, 2014b). Participants were video recorded and then invited to watch a video that allowed them to relive what they did while immersed and engaged in a climb. They were invited to use the video and audio recording to describe how they experienced the climb pre-reflectively without introducing any distorting interpretations and self-analysis of their performance and behaviour. Participants reported that they crossed ice tools to explore for existing holes in the icefall. Depending on the density, thickness, color, and sound of the ice, the climber was able to detect whether they should hook the hole or swing to better anchor the blade. The crossing of the ice tools thus allowed the expert climber to remain poised between hooking and swinging actions and attune to which of these actions the ice conditions invited.

In a study of trail running, phenomenological data was used to identify how runners experienced wearing different hydration systems (Rochat et al., 2019). Phenomenological data allowed the researchers to know when in a time-series the runner was noticing the hydration system. In particular, the runners reported that they felt the hydration system bouncing in an uncomfortable way in the flat and easy parts of the route and less on the technical parts of the route. The runners first-person experience allowed for the identification of a meaningful variable that helped to identify transitions in coordination dynamics, most notably the acceleration couplings between the hydration system and the runner (Rochat et al., 2019). Finally, in a study of rowers phenomenological data was used to identify perturbations in the in-phase coupling as rowers switch to out-of-phase coupling of their rowing actions. Phenomenological reports allowed the researchers to link these perturbations to factors such as wind, fatigue, waves and obstacles the rowers encountered such as other boats (Seifert et al., 2017).

The Skilled Intentionality Framework provides a bridge between an explanation of skilled actions in the terms of ecological dynamics and descriptions of the first-person lived experience of being a skilled agent like those that researchers in ecological dynamics are beginning to incorporate into their studies. Skilled intentionality refers to the selective openness and responsiveness to multiple relevant affordances in a concrete situation (Bruineberg & Rietveld, 2014; Rietveld, 2012). Skilled responsiveness to relevant affordances can be found in many activities traditionally thought to involve so-called “higher-order” cognition, such as planning, imagining and linguistically structured thinking. We see this for instance in the design practices of architects, which require architects to respond to affordances for a building that doesn’t yet exist. In the design process the architect is oriented towards and preparing for complexly structured possibilities for future action. They are continuously adjusting their creative acts in ways that they anticipate will advance the making of the building (Van Dijk & Rietveld, 2021). They do so in part through “switching between different ways of visualising the design, thus keeping the design ‘moving’” (Rietveld & Brouwers, 2017, p. 12). Skills are involved whenever an agent acts with knowledge and understanding, and all skilled action can and should be understood in terms of responsiveness to relevant affordances we believe. Thus, even cases of “higher-order” cognition that require knowledge and understanding can be understood in terms of the agent’s responsiveness to several relevant affordances simultaneously, or so the Skilled Intentionality Framework proposes (Rietveld et al., 2018).

The agent’s first person perspective of acting skilfully can be described in terms of relevant affordances standing out, inviting or soliciting the agent to act (Rietveld, 2008; Withagen et al., 2012). The ecological niche of an animal is best understood as a rich landscape of affordances offering an overabundance of possibilities for action (Rietveld & Kiverstein, 2014). The skilled individual is selectively open to this landscape and meets the landscape ready to act upon a selection of the possibilities available. This selection of relevant affordances will include some possibilities that are urgent because they relate to the agent’s immediate needs and interests (e.g. water when one is thirsty after a run). Other relevant affordances relate to what the agent needs to do in the future (e.g. meet a friend for drinks after work). At the same time as the individual is acting upon affordances in the here and now, they are also simultaneously ready for what to do next, and anticipating what they will be doing in the future.

The skilled individual is ready for a whole *field* of relevant affordances (Kiverstein et al., 2019). This field is structured in ways that reflect the individual’s needs and concerns with some affordances standing out as more important than others, and other relevant affordances forming the temporal horizon to those we are currently engaging with right now. The agent’s learning history and the skills and habits they have developed explain in part why in a given situation some of the affordances the environment has to offer stand out as relevant, inviting them to act, while others do not. The skills an individual has developed, sensitively attune them to relevant possibilities for action. A skilled architect for instance has acquired a complex repertoire of abilities for visualising and materialising possible designs, making models, finding the right materials, and finding the right craftspeople for making life-size mock-ups (Rietveld & Brouwers, 2017; Van Dijk & Rietveld, 2021). Based on these skills, certain affordances will stand out to them as to be acted on as they go about their work.

In order to be open to relevant affordances, an agent needs to attune to the information in the environment. Here we use “attunement” as it is sometimes used in ecological psychology to refer to the sensitivity to information that makes possible the perception of relevant affordances. Information exists by virtue of constraints. In their most general sense, constraints reduce the possible states a system can be in, and because of this reduction of possibilities, regularities occur between different aspects of the environment. Given these regularities, coupling to one aspect of the environment can be informative for what can be done and allow one to coordinate one’s activities with another (distal) aspect of the environment. In the standard case in ecological psychology, a pattern of light at a particular point in space lawfully specifies the availability of an affordance at a different point in space. Relaxing the requirement of lawful specification allows doing justice to (situated) normativity—the sensitivity to doing better or worse in a particular situation in the context of a given community’s socio-material practice. One can for example, do better or worse at kicking a ball, one might mistime one’s stepping behaviour and miss the ball completely. The informative regularities one makes use of are most of the time good enough but they do not guarantee success in coordinating one’s activities with the available affordances as would be the case if the information variables were lawfully specifying of affordances (see discussion in Chemero 2009, ch.6 and Bruineberg et al., 2019).

Among the regularities that we encounter in our socio-material practices are places and behaviour-settings such as cafes, supermarkets, libraries or swimming pools. They pre-structure the field of relevant affordances of the individuals that act in these settings (Barker, 1968; Bruineberg & Rietveld, 2014; Heft, 2018; Rietveld & Kiverstein, 2014). For example, the need to buy a ticket is relevant when travelling by train, but not when visiting a local café. In being ready to act in ways that are appropriate to a place one is ready for the whole complex and nested structure of affordances found in that familiar place or behaviour setting. The nested structure of the place (or what can be called a “place affordance”) allows the individual to have a grip on multiple possible actions at the same time just by having a grip on the nested structure of the familiar place or setting as a whole.

To maintain grip on many relevant affordances at the same time requires an individual to be ready for many action possibilities, and to change their openness to these possibilities when circumstances change. We will introduce the concept of “metastable attunement” to understand how this is possible. Skilled agents will often act in a metastable zone that allows them to keep their options open by remaining poised over multiple possibilities for action simultaneously. By remaining in a state of metastable attunement they can switch among affordances smoothly when something changes either on the side of the agent or in the environment. Although the environment may be unpredictable and even change randomly, this switching process is not a random process because it is guided by a history of past skilled engagement. Dealing with the messiness of everyday life requires finding a balance between the tendency to exploit skills existing in his or her repertoire versus the tendency to explore new solutions. To develop this idea further we need first to further explain the concept of metastable dynamics, and how the skilled agent makes use of what we are calling “metastable attunement” to remain poised over a field of multiple relevant affordances. We take up this task in the next section.

## Metastable attunement in agent-environment systems

As mentioned above, metastability refers in dynamical system’s theory to two competing tendencies: the tendency of the parts of the system to segregate and express their own intrinsic dynamics, and the tendency of the parts to integrate, and coordinate globally to create new dynamics. Kelso describes this concept in relation to coordination dynamics as follows:

In coordination dynamics, metastability corresponds to a regime near a saddle-node [...] in which stable and unstable coordination states no longer exist [...], but attraction remains to where those fixed points used to be. (Kelso, 2012, p. 913)

**Fig. 1:**
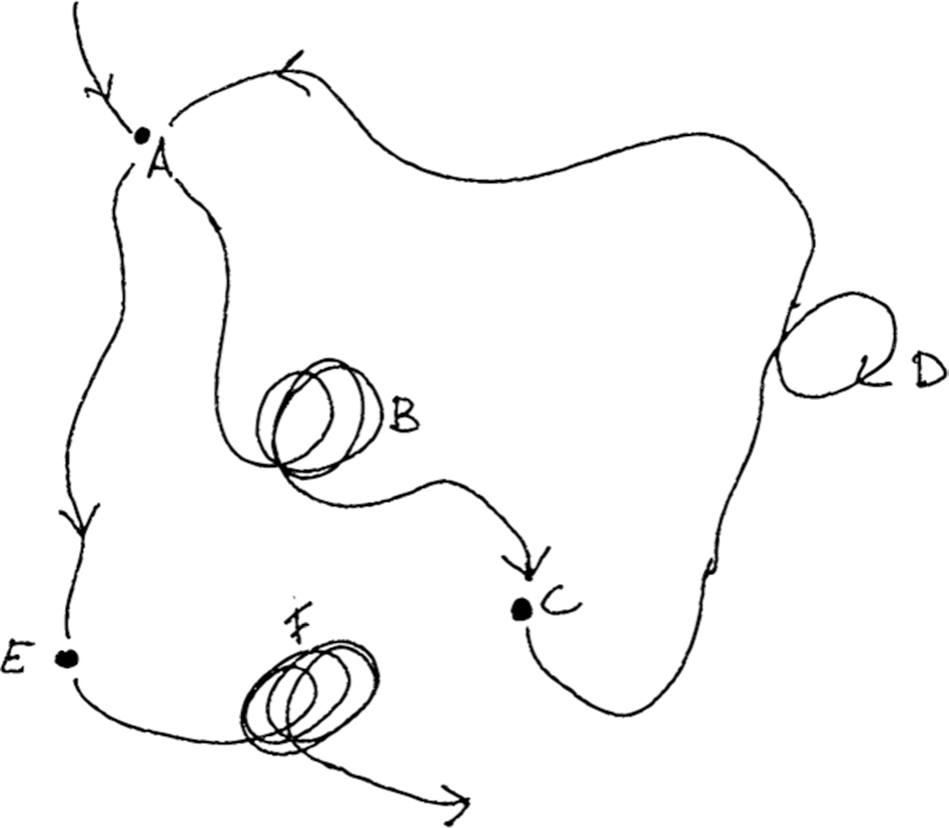
Schematic drawing of chaotic itinerancy. The system is attracted to certain places (B, D and F) but leave them after a (shorter or longer) period. The ongoing itinerant dynamics is characteristic of metastable systems (Figure adapted from Tsuda, 2001)

A saddle-node is a point in state space that is attracting coming from one direction, and repulsive in another direction: the middle of a horse’s saddle is the lowest (attracting) point when considered front to back, and the highest (repulsive) point when considered left to right. As a result, the system might be attracted to the vicinity of the saddle point, but will over time move away from it. Hence, metastable system transit between regions of state space spontaneously without the need of external perturbation. In a metastable system there is “attractiveness but, strictly speaking, no attractor" (Kelso & Engström, 2006, p. 172). Metastable dynamics are transient: there is a recurring creation and destruction of coordination that creates the impression of continuous instability while the attractor is constantly changing. However, for short periods, some stability can be observed, thus a metastable system does not reflect a complete lack of coordination. A schematic depiction of such dynamics can be found in Fig. 1. This transient dynamic stability is related to the phenomenon of intermittency, which defines a system equilibrium that is close to a critical state (i.e., a state at the margins of equilibrium, at which different states can co-exist) from which it can shift spontaneously from a coordinated to a disordered state.

Metastability can be contrasted with multistability. A multistable system has multiple stable patterns, or attractors. Due to constraints, or outside forces, working on the system, the system can switch between those attractors. In the context of agent-environment systems, multistability means that an agent has multiple behavioural patterns available for responding to relevant affordances. They switch between these behavioural patterns in ways appropriate to the demands of the situation (e.g. the adversity of your opponents in a sport competition), or when the capacities of the agent are challenged (due to e.g. fatigue or injury). Both multi- and metastability allow for degeneracy—for one and the same function to be achieved in different ways (Edelman & Gally, 2001; Price & Friston, 2002). Metastability is distinguished from multistability insofar as it is characterised by tendencies, not by co-existing stable motor patterns or coordinative structures. Thus in the boxing study we described in our introduction, the boxing striking patterns (jab, upper-cut, hook) rapidly transitioned in coordination with the fluctuations of the boxing bag. Metastability also allows the agent to rapidly switch from exploiting already familiar action possibilities to exploring novel possibilities when this is what the situation demands.

Metastability is a well-studied phenomenon in ecological dynamics. In one study for instance, researchers attempted to design a climbing environment allowing safe exploration within a metastable regime of performance (Orth et al., 2018; Seifert et al., 2015). The researchers designed three climbing routes by manipulating the hold orientation and the number of available edges for grasping. A horizontal-edge route was designed to allow horizontal hold grasping when the trunk face to the wall was observed. A vertical-edge route was designed to allow vertical hold grasping where the more challenging trunk side to the wall usually emerge in experienced climbers. Finally a double-edge route was designed to create a metastable regime of performance inviting both horizontal and vertical hold grasping. As a route with only vertical-edge holds was very challenging for novice climbers, the double-edge route would allow safe and functional exploration, as agents could both exploit their pre-existing behavioural repertoire (i.e., horizontal hold grasping pattern and trunk face to the wall) and explore new behaviours (i.e., vertical hold grasping and trunk side to the wall) (Orth et al., 2018; Seifert et al., 2015). The results indicate that metastable dynamics can be useful as perceptual-motor exploration appears ‘less risky’. Furthermore, the safety or stability provided by grip in the familiar kind of horizontal holds, had the consequence that the learner was more inclined to experiment in this zone.

There are important and often overlooked differences between skilled activities as they unfold in real life, and skill as it is investigated in the lab. In the lab the agent has a specific goal set by the experimenter. The situation in which the experimental participants are acting is well-defined in the sense that it is set-up so that the agent can perform actions that are self-contained, and isolated from the rest of the agent’s concerns. In real life, all sorts of unpredictable and unexpected things happen but typically people are able to smoothly adapt. Agents do not act on the basis of predetermined goals and intentions that map-out in advance the precise details of what they are going to do, nor are they following anyone’s instructions.^6^ The control of action doesn’t happen internal to the individual but unfolds in the agent’s coupling with the environment; i.e. in the whole agent-environment system (cf. Gibson, 1979). The short-term and small-scale events of neuromuscular systems *self-organise* into slower ecologically meaningful patterns of activity in the course of the agent’s dynamical coupling with an environment rich with affordances (Gibson, 1979; Reed & Bril, 1996). Meaningfully structured patterns of activity evolve over time in ways that are delicately tuned and adjusted to fit affordances. Moreover, the affordances available in an ecological niche form complexly nested structures such as places or behavioural settings (Barker, 1968; Heft, 2018). Thus skilled action in everyday life resists being carved up into self-contained, discretely existing situations with clearly defined goals (like those that experimenters artificially construct for participants in the lab). The patterns of behaviour affordances invite in a person’s life are intrinsically variable and open-ended in terms of the precise details of what happens (see eg. Van Dijk & Rietveld, 2021). There is enormous *variability* in the dynamics of agent-environment systems. No two situations are ever exactly the same. Think for instance of cycling through a busy city everyday and how the traffic conditions vary slightly each time you make the journey. This variability is difficult if not impossible to reproduce in the tightly controlled conditions of an experiment in the lab.

Metastable attunement makes possible flexible adaptiveness in an agent’s behaviour to the relatively stable constraints of the situation, and for flexible switching when the situation changes. Metastable attunement allows the agent to smoothly switch between exploitative sensitivity to affordances and explorative behaviour based on their attunement to the environment. We explain in the next section how the combination of flexibility and stability in behaviour that is a feature of metastable dynamics allows the agent to transit between established and relatively stable patterns of behaviour. This combination of flexibility and stability allows the agent to explore playfully in search of novel and innovative ways of achieving coordination with the environment when the agent senses this is what the situation requires (Seifert et al., 2013, 2016).^7^ We will argue that metastable attunement is necessary for adapting skilled action to the demands of the particular situation in real-life.

## Learning to exploit metastability in skilled action

The self-organising agent-environment system is able to rapidly switch from readiness to do one thing, to readiness to do another because the agent is perpetually in transition, poised over multiple action possibilities but ready to explore for novelty. So far in our paper we’ve described how a skilled agent is sensitively attuned to *how well they are doing* in their engagement with the environment. They are sensitive to whether they should “continue on a promising path” or self-organise so as to “jump to another attractor” (Dreyfus, 2007, p. 259). The possibilities to either exploit well-learned patterns or to explore in search of novel affordances are possibilities that we are suggesting derive from sensitivity to how things are going. The skill the agent has developed in the past allows them to exploit metastable dynamics so as to either make use of already well-learned patterns of behaviour and adapt them to the particular, unique situation. Alternatively they can make use of metastability to switch and explore novel and hitherto unexplored modes of engagement, allowing them to further improve their skill through learning.

Learning, as we will understand it, is the process of tuning perception and action systems to the ecological niche and the enormous variety of possibilities for action it provides in real life situations. Improvement in performance and thus learning happens when perception and action systems attune to new constraints or relevant affordances (Jacobs & Michaels, 2007). This process of attunement to the situation requires the agent to find patterns of behaviour that satisfy simultaneously constraints arising from the socio-material structure of the environment (in the case of human agents), and the biomechanics, physiology and morphology of the body.

In practice, learners often resist departing from already established behavioural pattern(s), as everyone knows who has tried to break with a bad habit. The tendency to leave or to maintain (i.e. to resist leaving) an existing coordination pattern relates to the *level of competition* (i.e. gap) between the to-be-learned pattern and the intrinsic dynamics (or past learning) (Zanone & Kelso, 1992). Learning can thus take at least two routes. What we will call the “bifurcation route” of learning corresponds to the emergence of a new pattern in the coordination dynamics and reflects a *selection *via* instability* (Kelso, 2012). A “bifurcation” reflects an abrupt qualitative change as a new attractor states emerges and is stabilised into the landscape. The second route to learning described as “shift learning” corresponds to the refinement of the layout of attractors in the landscape (Kelso, 2012). In the case of the *shift* route to learning, coordination dynamics do not increase the number of attractors but are only refined to match the environmental constraints. Kelso (2012) called this selection of new behavioural pattern ‘*selection *via* matching’*. This matching corresponds to gradual shifts in the direction of the to-be-learned pattern without exhibiting any instability nor higher stability.^8^

It is not only through bifurcation that agents can learn a new pattern of behaviour. A third route might pass by a period of intermittency reflecting metastability (Kelso, 2008, 2012). Recall how metastability refers to a dynamical regime that is neither stable nor totally unstable (Kelso, 2012), allowing learners to build upon their existing stable patterns of behaviour by exploring for novel affordances. In a metastable regime, the past learning of an agent provides the relatively stable scaffolding for exploratory behaviour and innovation (Sporns & Edelman, 1993). Exploration through a metastable regime may occur over a more or less extended period of time. The agent may transition intermittently between the existing stable patterns and emergent possibilities until a new stable coordination pattern stabilizes (Chow et al., 2008; Teulier & Delignières, 2007).

**Fig. 2:**
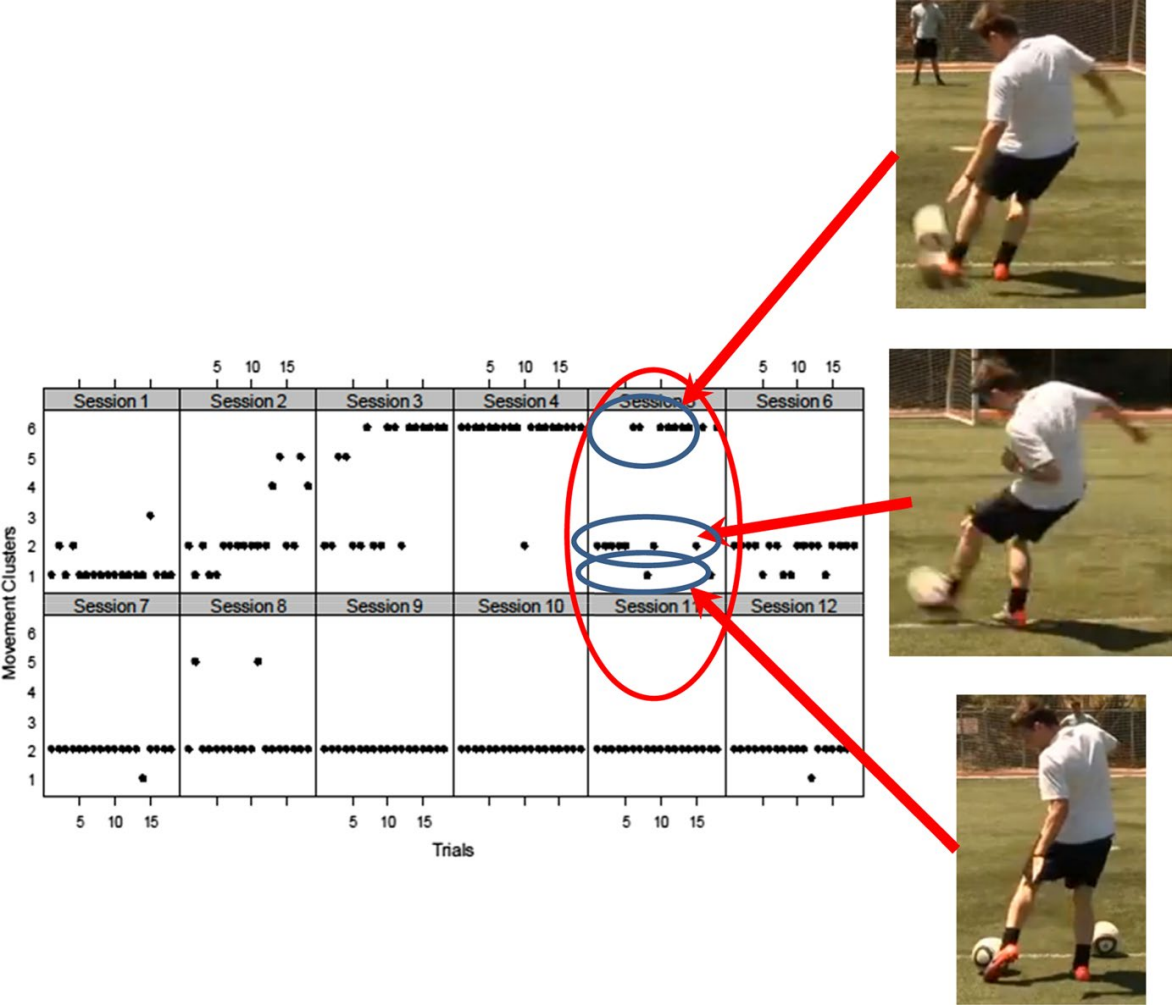
This figure illustrates how a learner can start the learning process (session 1) with one stable pattern (cluster 1) and then switch to another pattern (cluster 2) at session 2. In session 3 the learner starts to exhibit metastability by switching between clusters 2, 5 and 6. Pattern 6 briefly stabilises in session 4, to then exhibit again a metastable regime of performance in session 5 (where intermittency exists between pattern 1, 2 and 6). Finally the learner stabilizes in pattern 2 from session 7 to 12 (Figure adapted from Chow et al., 2008)

Thus in Chow et al., 2008, the football player navigated between three states (patterns 1, 2 and 6) without being locked into any of these states (Fig. 2). The player navigates between a stable state (pattern 2) present at the beginning and at the end of the learning process, and unstable states (pattern 1 and 6) that temporarily attract the player. Pattern 1 is only present at the beginning of the learning process and pattern 6 is not present at the beginning but is present at the end of the learning process. This learning process is metastable, rather than multistable, because the states are only transient. The existing coordination pattern is used as a platform to explore toward more skilled behaviours. At the same time, the emerging novel behaviour remains only relatively stable, allowing learners to fall back on existing behaviours as back up (Teulier & Delignières, 2007).

**Fig. 3:**
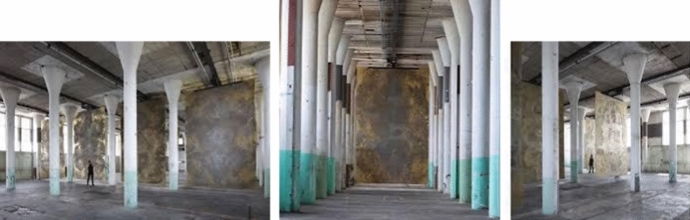
Pictures of the *Still Life* installation. Photos: Jan Kempenaers

**Fig. 4:**
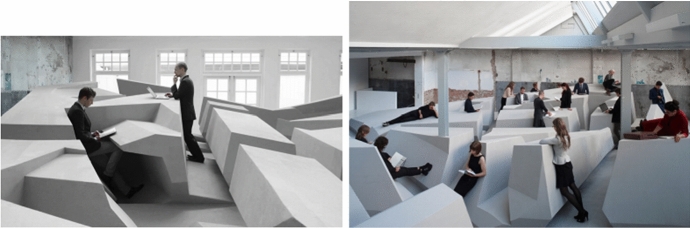
*The End of Sitting*. Left: use of two different positions in *The End of Sitting* landscape. Still from the film *The End of Sitting 1:1*. Right: overview of *The End of Sitting*. Photo: Jan Kempenaers

Metastable attunement allows the agent to rapidly switch from exploiting already familiar action possibilities to exploring for novel possibilities when this is what the situation demands. Skilled action as it occurs in real-world settings is characterised by a combination of stability and flexibility (Seifert et al., 2013; Warren, 2006). For instance, if the situation is one in which an icefall climber must anchor his ice axes in the ice surface, he can demonstrate functional stability such as “swinging” the ice axes against the icefall exploring for specific anchorages. But if the icefall already provides support in the form of existing holes in its structure, an ice climber can also demonstrate functional flexibility by “hooking” the blade of the ice axes into these existing holes in the icefall, affording support on the ice surface (Seifert et al., 2014a, b). Here the climber is using the hooking movements to explore the quality of existing holes as possible points of anchorage. The swinging action reflects the ‘exploitation’ of existing patterns in the dynamics of the joint climber-icefall system while the hooking action allows for exploration of new dynamics that emerge through the coupling of the blade and icefall. These behavioural patterns of hooking or swinging the ice axe are stable in the sense that they are consistent over time, resistant to perturbations and reproducible in that a relatively similar pattern may emerge under different task and environmental constraints. However, they are also metastable and not multistable because the climber spontaneously switches to hooking based on their attunement to opportunities the icefall affords.

Metastable dynamics make it possible for the agent to leave stable behaviours in order to explore new possibilities but also to come back to his or her initial behaviours. In sum, when metastability occurs, the agent navigates between two opposite tendencies (the tendency to preserve and to exploit stables behaviours and tendency to explore new solutions) in order to fit what the situation demands. The complex unpredictable environment people deal with in real life requires the agent to be poised between these two possibilities as they adapt what they are doing to the demands of the particular situation.

## How to investigate metastable attunement in real life?

How might a situation be structured so that agents are drawn to act skilfully in a metastable zone? In this section, we use examples from art and architecture and sports science to show how the environment might be designed to induce and facilitate zones of metastable attunement. Recall that in the previous section we distinguished between learning in a multistable regime and learning in a metastable regime based on the relation between the to-be-learned pattern of behaviour and the agent’s intrinsic dynamics (their past learning). We have seen above how metastability occurs within transition points in performance. These transition points in performance can be used as zones that facilitate the improvement in skills.

### Investigating metastable attunement through art

Merleau-Ponty (1945/2002) described how in an art gallery, a person may be drawn into a vantage point on a painting from which the painting can be best seen. Where the example from Merleau-Ponty focuses on one single affordance, in this paper we zoom out to include the way in which an agent is situated in a broader context and practice. Skilled agents tend towards grip on multiple affordances simultaneously (the painting, the other visitors, the closing of the museum later that afternoon) and are sensitive to the particular norms in the form of life (touching the painting is prohibited). At some point, something else, perhaps a different painting in the art gallery, shows up as relevant and the agent switches to a new trajectory. Skilled agents are metastably attuned to a multiplicity of affordances. We present the art installation *Still Life* as a materialization of the concept of metastable attunement in order to make this concept tangible and experienceable.

*Still Life* is a large site-specific art installation in a new space for contemporary art in Amsterdam that had an earlier life as a place of weapon industry. In the former munitions factory Het HEM in Zaandam in the Netherlands, millions of bullets were produced during the Cold War for the use of NATO soldiers worldwide. The source material for bullet production has been melted down and cased into four heavy brass plates for the artwork *Still Life*. The large plates, each weighing 1500 kg, and spanning 5.30 m by 3.30 m, are placed parallel between the columns and slowly and rhythmically move between the columns, 70 m across the factory hall. Combined, they open and close the perspective of this immense space. The viewer is forced to continually re-attune themselves with the work and the space, as the heavy brass plates move slowly through the space (Fig. 3).

*Still Life i*s an artwork that embodies in multiple ways the principle of metastable attunement. First, just like in Merleau-Ponty’s example, a visitor is drawn towards the plates. But as she approaches an optimal position, the plate itself looms closer and she is repelled by the big mass coming towards here. A stable “optimal” point is therefore never reached, but continuously tended towards. Second, the casted plates have a front side and a back side and are situated in a space. It is not just the plates that afford visual exploration, but also the way they are situated in and move across the factory hall. Third, the installation consists of 4 plates moving simultaneously. As one wanders away from one plate, one comes across the trajectory of the next one. This increases the possibility of flexibly switching from one perspective to another. The multiple moving plates also require a certain circumspective awareness. When stepping back from one plate, the visitor has to be careful not to step in the vicinity of another moving plate. As a result of these dynamics, the trajectory of a visitor is somewhat reminiscent of the chaotic itinerancy trajectories of Tsuda (2001) (Fig. 1).

### Constraint manipulation

We suggest that the development of skill can be guided in pedagogy by manipulating constraints that fall either on the side of the organism (internal dynamics) or on the side of the environment (external dynamics) so as to draw the agent into a metastable zone. It should be noted that while we allow these factors can be separated formally for the sake of analysis and modelling, in practice they are tightly coupled and exert reciprocal causal influence on each other. They combine to form the context in which skilled action unfolds.

Constraint manipulation can be used to amplify or to hide relevant affordances of the landscape of affordances. This approach to learning views the factors that influence learning as constraints, which guide the acquisition of movement coordination and control by setting boundaries within which exploration of the perceptual motor workspace is encouraged (Newell, 1991, 1996). According to Newell (1986), constraints can be classified into three distinct categories:^9^

*Organismic constraints* refer to characteristics of individual performers, such as genes, height, weight, muscle-fat ratios, connective strength of synapses in the brain, cognition, motivations and emotions. For example, women typically have a different ratio and repartition of fat mass and strength than men, constraining their buoyancy in the water, their stroke rate/stroke length ratio and their motor coordination in swimming (Seifert et al., 2011). These unique characteristics represent resources that can be used to solve movement problems or limitations that can lead to individual-specific adaptations by a performer.

*Task constraints* are usually more specific to particular performance contexts than environmental constraints and include specific performance goals, rules of a specific sport, equipment and implements to use during a physical activity, performance surfaces and boundary markings. A particularly significant task constraint is the information present in the learning environment. Gibson (1979) argued that neurobiological systems are surrounded by arrays of energy flows, that can act as information sources (e.g., optical, acoustic, proprioceptive) to support decision-making and movement organization during goal-directed activity. For instance, recent investigation in swimming attempted to highlight how a dynamic water flow, such as swimming in a flume, in an ocean or in a river involved a different behaviour in comparison with swimming in a pool where water is in a quasi steady state (Guignard et al., 2017). Guignard and colleagues postulated that it is useful to train swimmers in a flume to educate their attention to the dynamics flow as swimmers’ actions impact the motion of water particles in a circular manner and, in return, the fluid motion will impact their future perceptions and actions.

*Environmental constraints* can be physical in nature, such as ambient light, temperature or gravity. For example, gravity is a constraint that skilled climbers play with by swinging their body like a pendulum or by rolling their trunk like a door, most notably when the target hold is beyond the arm length (for more details, see Cordier et al., 1996; Seifert et al., 2015). Manipulation of the environmental constraints could be more or less important in order to push agent out of their comfort zone leading to metastable regime of performance. By radically restructuring the available affordances in a certain place it is possible to generate behavioural change. With another artwork the possibility of creating a zone of metastable attunement has been explored by RAAAF|Barbara Visser. Their art installation *The End of Sitting* offers a landscape of affordances for supported standing, in the sense that surfaces afford working in several non-sitting postures (e.g. standing, leaning, lying) (Rietveld, 2016; Withagen & Caljouw, 2016). One could see this landscape as a material field of promoted action in the sense that it amplifies possibilities for supported standing and does not offer possibilities for sitting (Fig. 4).

*The End of Sitting* installation is an artefact for the promotion of supported standing. The artwork questions the predominance of sitting behaviour in our society. Its title makes people aware of their habitual sitting behaviour and the possibility for behavioural change. The *End of Sitting* installation goes from lower to higher so as to offer possibilities for supported standing to people with different body lengths as well as easier and more challenging possibilities for supported standing. By offering a large variety of positions, people are free to select the position that is suited to support their current activity and changing capacities (e.g. certain muscles get tired after standing in a given position which will then make other affordances for supported standing, leaning or hanging stand out as relevant) (Withagen & Caljouw, 2016). *The End of Sitting* is an example of a metastable zone in real life as switching between positions happens because muscles used in any position will get tired and will make people switch to different places in the landscape.

This approach shown by The End of Sitting could be further developed in several directions. Educators and instructors, architects and designers could induce a metastable zone of performance by manipulating constraints and designing a *field of promoted actions* (Reed & Bril, 1996). Reed and Bril (1996) defined the "*field of promoted action*" as a field where the experimenter introduces objects, places and activities designed to promote engaging with some affordances, and discourages engaging with others, which could be achieved both by amplifying and by hiding (masking) environmental properties. From there, Reed and Bril (1996) and (Bril, 2002) outlined four characteristics of a field of promoted action: (1) an action may be more or less encouraged or prohibited, (2) certain artefacts (tools use) and some affordances may be more or less widely available in the agent’s environment, (3) in any society there are rules on the roles to be performed by different agents in different situations, and the objects that should then be used, and (4) the organization varies depending on age, developmental level, and skill for a particular behaviour at a given time.

### Designing rich landscapes of affordances

One can also promote *exploration* by designing rich and attractive landscapes of affordances. The main reason to design rich landscapes of affordances is to promote *creativity* and *innovation* through exploratory behaviours. One might think that sanitising the landscape of affordances would offer more stability, but this short-term effect would mainly narrow behavioural flexibility and ignore the diversity and variability of the ecological niche. Take for example the culinary monoculture of eating fast food and drinking soda. While providing stable and easy affordances for eating almost anywhere in the world, it completely ignores the richness and diversity of local food, resources and recipes. Exploration is a basis of creativity. We hypothesise that designing a metastable zone of performance could help to enrich the behavioural repertoire.

In sport science, there are ample examples of inducing a metastable zone to explore the behavioural repertoire. As an example in perceptual-motor systems, Seifert et al. (2014a, b) showed that when a long gliding phase was requested with the arm stretched forward during a front crawl swimming task, unexpected behavioural leg beat-kicking patterns can emerge. Whereas two, four and six leg beat-kicking patterns for one are cycle are the most common patterns observed in competition, in this study eight to ten leg beat kicking per arm cycle was used to compensate the arm glide (Seifert et al., 2014a, b). These findings revealed how swimmers were able to use perceptual-motor system degeneracy to overcome an atypical constraint on their stable leg kicking patterns in order to satisfy an imposed task requirement. By doing so, swimmers also learnt to vary the timing of their leg kicking in generating propulsion as well as ensuring body balance. Thus, by moving the perceptual-motor system out of its comfort zone and proscribing existing patterns, practitioners can create a richer landscape of affordances as it enlarges the initial function that the system can achieve (Seifert et al., 2016). Nevertheless, the challenge for practitioners is to set realistic boundaries as no one would like to restrict exploration or cause either unsafe exploration or even injury.

### Skilled performance in a metastable zone

We have used the term “metastable zone” to refer to a region in the phase space of an agent-environment system in which the agent is poised between exploiting affordances they have already mastered, and going beyond their existing repertoire in exploring for novel affordances.^10^ Coaches and educators would argue that training in a metastable zone can improve performance and help players to be more skilled (Seifert et al., 2013). How could a learning situation be structured so that agents act in a metastable zone? In the following study, a metastable zone of performance was induced in cricket batsmen (Pinder et al., 2012). The bowler in cricket typically attempts to induce uncertainty in the batter about whether to step forward or back to make contact with the incoming ball. This experiment sets out to test, through movement-timing, how stable the patterns of movement were in the batsman under different constraints such as body-scaled ball pitching location. The experimenters expected to find the emergence of a metastable region—a pitching location—that would require the batsmen to transit flexibly between two movement responses (i.e. moving forward or back). They found that batsman did not show any stable preference for the bouncing zone on the pitch. They did not show any preferred or stable behaviour while hitting the ball. When the ball rebounded 7.5 m from the batsmen, they sometimes moved forward and sometimes backward in order to strike. The batsman’s movements, contact time, and the direction of the batting shot showed great variability when the ball was delivered into this zone, suggesting a metastable regime of performance. In contrast, these parameters showed high stability when the ball bounced in zones closer to, or more distant from the batsmen. Skilled batsman could thus be considered as acting in a metastable zone of performance (Pinder et al., 2012). The learning situation in which a metastable zone is found, can be used both to explore new behaviours and to promote behaviour flexibility as an answer to the constraints of the situation. All this is well known in the literature on sports science, but the considerations outlined in this section point to applications in design, architecture and art.

## Conclusion

In real life all sorts of unpredictable and unexpected things happen but typically people are smoothly able to adapt. We’ve argued that metastable attunement is necessary to deal creatively with the messy and unpredictable situations in which everyday skilled action takes place. Metastable attunement captures the peculiar mix of stability and flexibility that is required for dealing with the enormous variability offered by everyday life.

In a number of recent applications of ecological dynamics, the behavioural data was complemented by reports on the phenomenology of skilled action to better understand how agents experienced ecological context of performance (Rochat et al., 2019; Seifert et al., 2014a, b, 2017). The notion of metastable attunement developed in this paper has roots in both phenomenology and ecological dynamics. We therefore think it can serve as a bridging concept between the third-person and first-person investigation of skilled action.

The reason skilled action in everyday life has proven so difficult for cognitive scientists to explain is that they have not (yet) been able to investigate behaviour at the scale of the whole agent-environment system in real-life situations; the agent’s coordination with multiple nested affordances that are enacted simultaneously but unfold on multiple time-scales. Metastable attunement, we have suggested, allows agents to remain poised between exploiting affordances they are already familiar with because of their past history of skilled engagement and exploring for novel affordances that allow them to creatively improvise and expand their repertoire of skills. We have finished up by suggesting ways in which metastable attunement might be investigated in real life given the challenges of studying it in the lab. We’ve described how situations might be structured in art and architecture and in sport science so as to draw agents into situations with which they can metastably attune.

An important question for further research is how to design metastable zones that invite safe exploration, i.e. exploration with a back up. People will typically not explore the landscape of affordances if the consequences of doing so could lead to injury or death. Therefore, metastability should not bring agents to an uncoupling or instable regime, but to search and transit toward new functional behavioural patterns from a position of strength. Metastable attunement allows the organism to be ready for multiple possibilities simultaneously and to gradually shift or to abruptly transit between behavioural states when unexpected events or disturbances happen either internal to the individual (e.g., exhaustion, injury) or external of the individual in the environment (e.g., the warm and humid weather that potentially destabilises athletes at the 2021 Olympics in Tokyo). Placing agents in metastable region is challenging and makes it important to set boundaries allowing both exploration of new affordances for the agent and familiar affordances as back up that could allow one to fall back on using existing behavioural pattern (Seifert et al., 2016; Teulier & Delignières, 2007). Such zones of metastable attunement can provide agents with a safe basis for exploration and creative improvisation, which allows them to make progress in skill learning and to find their way to novel and unconventional affordances.

## Footnotes

1 For important earlier work on metastability and multistability in dynamical cognitive science see Tsonis (1992), Kelso (1995, 2012), Friston (1997), Bressler and Kelso (2001) and Tognoli and Kelso (2014).

2 This ecological conception of attunement is sometimes characterized in terms of “resonance”. The tuning of perceptual systems to environmental information is then understood analogously to the workings of a radio: in tuning in to a specific frequency, the radio extracts information from a wealth of background noise (Gibson, 1979, p. 249; Raja, 2020). This metaphor leaves a lot to be desired. As Heft has noted: “a tuning fork resonates, and indeed, cannot help doing so ceteris paribus. But an individual selectively engages the environment […] Because knowing processes are marked by an individual selectively engaging the environment, a term with a more intentional connotation may better direct the thinking here. In this respect, attunement would seem to be more suitable.” (Heft, 2001, p. 366).

3 The idea that our experience *is* our active engagement with the world is a basic commitment of the enactive approach to embodied cognition (Varela et al., 1991).

4 This current paper is very much in agreement with a recent proposal to bridge ecological dynamics and skilled intentionality (Araujo et al., 2019). Araujo et al. (2019) make this bridge in the context of sports science. We use insights from ecological dynamics in sports science and phenomenology to apply it to the broad range of situations encountered in *everyday life*. In the latter part of the paper, we point to applications in design, architecture and art.

5 Interestingly this first-person perspective (in agreement with the enactive approach) provides descriptions of skills in everyday life that complement empirical questions that arise within the ecological dynamics framework. See for example Araujo et al. (2005) and the more recent studies by Rochat et al. (2019) and Seifert et al. (2017) we discuss in Sect. 2 of the paper.

6 Of course, agents *do* have personal-level goals. The point here is that attunement to affordances does not (necessarily) align with those goals. The question how personal-level goals can be made aligned with attunement to affordances is an important one (see Brancazio & Segundo-Ortin, 2020).

7 An anonymous reviewer pointed us to self-organized criticality as discussed in Juarrero (1999), Van Orden and Holden (2002) and Van Orden et al. (2003). They saw parallels between our notion of metastable attunement and the literature in ecological psychology concerned with self-organised criticality. “Criticality allows an attractive mix of creativity and constraint. It creates new options for behavior and allows the choice of behavior to fit the circumstances of behavior” (Van Orden et al., 2003, p. 333). This is in agreement with metastable attunement as we described it above. However one important difference is that we don’t only consider that the agents stay near critical states, but, instead, that they continuously adapt between two opposite tendencies, the tendency of integration that is the preservation of intrinsic dynamics and the tendency of segregation. Second, our concept of metastable attunement aims to capture the phenomenology of attuning to the relevant affordances of the environment, while remaining poised to change our openness when necessary. Discussions of self-organised criticality by contrast explore the link between intentional control as understood in ecological psychology and physics. Our concept of metastable attunement aims to build a bridge between dynamical systems theory, ecological psychology and phenomenology. This is not work that the notion of self-organised criticality can do on its own.

8 According to Kostrubiec et al. (2006), the *bifurcation* route of learning seems to show greater persistence of a learned behaviour in memory, whereas these authors claim that the shift route of learning reflects flexibility and short term adaptation, but is prone to forgetting.

9 For Newell (1986), the interaction between organismic, environmental and task constraints specifies an optimal pattern of coordination for the agent. An agent can come to learn this pattern of coordination. Performance can then be characterized as the individual interacting with the task and environmental constraints.

10 The metastable zone refers to a region of the phase space of an agent-environment system. In specific cases, this will correspond to a particular relation between agent and environment. In the boxer example mentioned above, the metastable zone corresponds to a distance between the boxer and the boxing bag of 0.6 times the boxer’s arm length.
